# Wnt6 maintains anterior escort cells as an integral component of the germline stem cell niche

**DOI:** 10.1242/dev.158527

**Published:** 2018-02-01

**Authors:** Xiaoxi Wang, Andrea Page-McCaw

**Affiliations:** Department of Cell and Developmental Biology and Program in Developmental Biology, Vanderbilt University School of Medicine, Nashville, TN 37240, USA

**Keywords:** *Drosophila*, Wnt signaling, Germline stem cells, Oogenesis

## Abstract

Stem cells reside in a niche, a local environment whose cellular and molecular complexity is still being elucidated. In *Drosophila* ovaries, germline stem cells depend on cap cells for self-renewing signals and physical attachment. Germline stem cells also contact the anterior escort cells, and here we report that anterior escort cells are absolutely required for germline stem cell maintenance. When escort cells die from impaired Wnt signaling or *hid* expression, the loss of anterior escort cells causes loss of germline stem cells. Anterior escort cells function as an integral niche component by promoting DE-cadherin anchorage and by transiently expressing the Dpp ligand to promote full-strength BMP signaling in germline stem cells. Anterior escort cells are maintained by Wnt6 ligands produced by cap cells; without Wnt6 signaling, anterior escort cells die leaving vacancies in the niche, leading to loss of germline stem cells. Our data identify anterior escort cells as constituents of the germline stem cell niche, maintained by a cap cell-produced Wnt6 survival signal.

## Abstract (precis)

**Summary:** In *Drosophila* ovaries, anterior escort cells, maintained by Wnt signaling, form a crucial component of the stem cell niche by promoting BMP signaling and anchoring GSCs through DE-cadherin-based junctions.

## INTRODUCTION

Adult tissues are maintained by stem cells that self-renew and differentiate into functional cells. Stem cells reside within a specialized microenvironment known as the niche, and their self-renewal, numbers and activities are regulated by extrinsic cues from the niche (Li and Xie, 2005). Understanding the niche structure is fundamental to harnessing stem cells in applications such as regenerative medicine. The cellular organization of the stem cell niche is complex, and it can include stem cells themselves, their progeny, nearby mesenchymal cells or stromal cells, muscles, extracellular matrix, and distant sources within or even outside the tissue (Rezza et al., 2014). How different niche components interact with each other remains elusive.

Studies on *Drosophila* ovarian germline stem cells (GSCs) have provided an archetypal example of a stem cell niche composed of adjacent support cells. In the *Drosophila* ovary, two or three GSCs are located at the apex of each ovariole in a structure known as the germarium. GSCs form direct contact on their anterior side with a cluster of five to seven disc-shaped cap cells via adherens junctions. This anchorage is essential for GSC self-renewal (Song et al., 2002). Furthermore, cap cells secrete bone morphogenetic protein (BMP) ligands including Decapentaplegic (Dpp) and Glass bottom boat (Gbb) to repress differentiation of GSCs (Liu et al., 2010; Song et al., 2004; Wang et al., 2008; Xie and Spradling, 1998, 2000). As a GSC divides, it produces a self-renewing GSC daughter that remains in contact with cap cells, and a cystoblast daughter positioned away from the niche. Without continuous BMP signaling, the cystoblast differentiates into a germline cyst and eventually an egg (Xie and Spradling, 1998). For these reasons, the cap cells are considered to be the GSC niche.

**Fig. 1.**
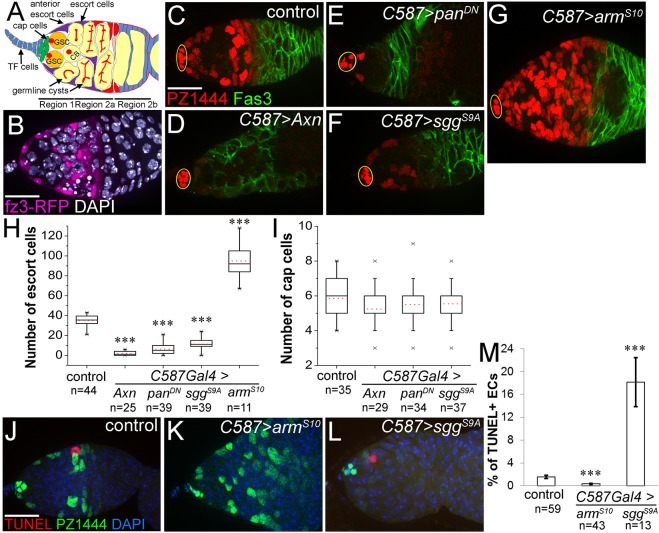
**Wnt signaling in escort cells promotes their survival.** (A) Schematic of the germarium. Germline stem cells (GSCs, orange) contact cap cells (green) anteriorly and anterior escort cells (purple) laterally. GSCs and cystoblasts (CB) are undifferentiated germline cells, and they contain a specialized organelle termed a ‘spectrosome’ (red) for its spherical morphology, which grows and branches into a ‘fusome’ in differentiated germ cells. TF, terminal filament. (B) Wnt signaling is active in escort cells as revealed by the *fz3-RFP* activity reporter (magenta). (C-G) Altering Wnt signaling in escort cells (with *C587Gal4*) controlled the number of escort cells. *PZ1444* (red nuclei) expresses *lacZ* in all escort cells and in cap cells (circled), visible as a cluster of cells at the anterior tip of the germaria with bright staining. Reducing Wnt signaling by overexpression of *Axn* (D), *pan^DN^* (E) or *sgg^S9A^* (F) resulted in a dramatic decrease in escort cell number. Conversely, hyperactive Wnt signaling caused by overexpressing *arm^S10^* increased the number of escort cells (G). Flies with *C587Gal4* and *tubGal80^ts^* were raised at 18°C, switched 1-2 days after eclosion to 29°C, and dissected 7 days (C-E,G) or 10 days (F) after temperature switch. (H,I) Box plots showing the number of escort cells (H) and cap cells (I) per germarium in the conditions shown in C-G. Mean values are shown as red dotted lines. (J-L) TUNEL staining identifies apoptotic escort cells, labeled by *PZ1444*. Compared with control (J), Wnt activation in escort cells decreased the rate of apoptosis (K), whereas inactivating Wnt signaling in escort cells increased apoptosis (L). (M) Percentage of escort cells that are TUNEL positive in the conditions shown in J-L. Absolute numbers are given in Fig. S1. Error bars indicate s.e.m. ***P<0.001 (Student's *t*-test). *n* indicates the number of germaria counted for each experiment. ECs, escort cells. Scale bars: 20 µm.

Escort cells are a population of 30-40 squamous cells that line the basement membrane of the anterior half of the germarium, and they extend cytoplasmic processes to encase each GSC, cystoblast and developing germline cyst (Fig. 1A) (Morris and Spradling, 2011). Escort cells play an essential role in germline differentiation, as many studies have shown that escort cell disruptions result in an accumulation of undifferentiated, stem-like germline cells (Eliazer et al., 2014; Hamada-Kawaguchi et al., 2014; Jin et al., 2013; Kirilly et al., 2011; Liu et al., 2010; Luo et al., 2015; Ma et al., 2014; Mottier-Pavie et al., 2016; Mukai et al., 2011; Rangan et al., 2011; Schulz et al., 2002; Upadhyay et al., 2016; Wang et al., 2015, 2011; Xuan et al., 2013). Over the last decade, scattered observations have suggested a role for unspecified escort cells in maintaining GSCs (Rojas-Ríos et al., 2012; Wang et al., 2011), but this role has not been probed in depth.

In this study, we demonstrate that anterior escort cells, which contact the GSCs, are essential for GSC maintenance. We find that, like cap cells, the most anterior escort cells anchor GSCs through DE-cadherin-based junctions, and these anterior escort cells produce Dpp ligand necessary for full-strength BMP signaling in GSCs. Furthermore, these anterior escort cells are maintained specifically by cap cell-secreted Wnt6 ligands: when Wnt6 is knocked down in cap cells, anterior escort cells frequently die and are not replaced, resulting in a loss of Dpp signaling and GSC loss from the niche. Altogether, our data provide direct evidence that anterior escort cells are an essential cell type within the stem cell niche, and they indicate that cap cells maintain anterior escort cells in the niche by promoting anterior escort cell survival through Wnt6 signaling.

## RESULTS

### Wnt signaling is required to maintain escort cell number

In the germarium, Region 1 contains mitotic germ cells, i.e. GSCs, cystoblasts, 2-, 4- and 8-cell cystocytes, whereas Region 2a contains 16-cell cystocytes (Fig. 1A). Escort cells are squamous somatic cells distributed throughout Region 1 and Region 2a up to the follicle stem cells (FSCs), and they encase germ cells at different stages until they become encapsulated by follicle cells in Region 2b (Fig. 1A). Our previous study showed that Wnt signaling in FSCs promotes their proliferation (Wang and Page-McCaw, 2014; see also Sahai-Hernandez and Nystul, 2013; Song and Xie, 2003; Vied et al., 2012). Interestingly, the pattern of a Wnt-signaling activity reporter, *fz3-RFP* (Wang and Page-McCaw, 2014) suggested that, in addition to FSCs, escort cells also exhibit active Wnt signaling (Fig. 1B). No *fz3-RFP* signal is evident in germline cells, consistent with reports that Wnt signaling is not required in the germline in the germarium (Song and Xie, 2003).

To investigate its function, we impaired Wnt signaling in escort cells by overexpressing *Axin* (*Axn*) or a constitutively active form of the GSK3β homolog *shagg*y (*sgg^S9A^*) (Hazelett et al., 1998), two components of the β-catenin destruction complex, or by overexpressing a dominant-negative form of *pangolin* (*pan*, also known as TCF) (van de Wetering et al., 1997). Adult-specific escort cell expression was induced with the *C587-Gal4* driver and *tubGal80^ts^* (Kirilly et al., 2011), and escort cells were labeled by the *PZ1444* enhancer trap, which also labels cap cells (Xie and Spradling, 2000). Control germaria have ∼35 escort cells, dispersed across the anterior half of the germarium with triangle-shaped nuclei labeled by *PZ1444* (Fig. 1C,H). Inhibiting Wnt signaling in escort cells by overexpressing *Axn*, *pan^DN^* or *sgg^S9A^* dramatically decreased the number of escort cells (Fig. 1D-F,H). *Axn* overexpression caused the most severe phenotype, with only approximately two escort cells remaining, whereas with *sgg^S9A^* overexpression ∼11 escort cells remained (Fig. 1H). Inversely, activating Wnt by overexpressing a constitutively active form of *armadillo* (*arm^S10^*) increased the mean number of escort cells from 35 to 95 (Fig. 1G,H). Although cap cells are also labeled by *PZ1444*, they are easily distinguished by their location and morphology as a cluster of five to seven disc-shaped cells at the anterior tip (circled in Fig. 1C-G), and cap cells were not altered by inhibiting Wnt signaling in escort cells (Fig. 1C-F,I). To investigate whether Wnt signaling was required for escort cell survival, terminal deoxynucleotidyl transferase dUTP nick end labeling (TUNEL) staining was performed. Decreasing Wnt signaling by overexpressing *sgg^S9A^* or *Axn* significantly increased the TUNEL-positive escort cells, both by number and percentage of total escort cells per germarium (Fig. 1J,L,M, Fig. S1). In contrast, increasing Wnt signaling with *arm^S10^* decreased the percentage of apoptotic escort cells (from 1.53±0.29% in control to 0.33±0.10% in *arm^S10^* overexpression, *P*<0.001) (Fig. 1K,M). This decrease in the percentage of apoptotic escort cells did not reflect a change in the number of apoptotic cells (Fig. S1) but rather the nearly 3-fold increase in total escort cell number (Fig. 1H), indicating that Wnt signaling also controls escort cell production. Similar results were generated by Wang et al. (2015). Thus, Wnt signaling is required for escort cell survival.

### Wnt signaling in escort cells regulates germline stem cell maintenance

**Fig. 2.**
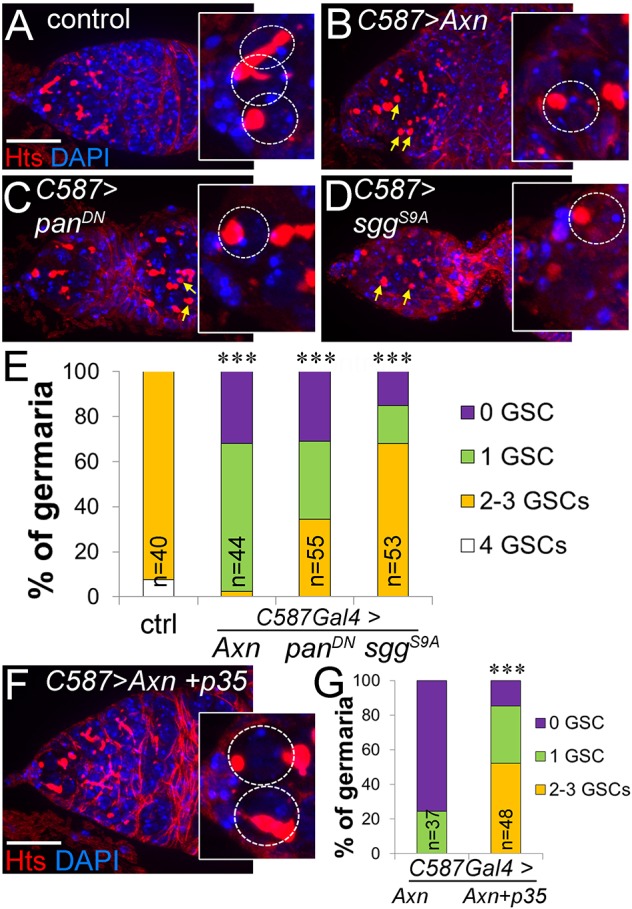
**Wnt signaling maintains germline stem cells by promoting escort cell survival.** (A-D) Loss of Wnt signaling in escort cells by overexpression of *Axn*, *pan^DN^* or *sgg^S9A^* caused a decrease in the number of GSCs (outlined by dashed circles in insets). GSCs were identified by anti-Hts staining (red) and their attachment to the anterior cap cells. Anti-Hts also labels excess undifferentiated germline daughter cells (yellow arrows), not attached to cap cells. (E) Quantification of GSC number in the conditions shown in A-D. All flies contained *C587Gal4* and *tubGal80^t^*^s^ and were switched from 18°C to 29°C upon eclosion for 1 week before dissection, to express ectopic genes in adult escort cells only. ***P<0.001. (F,G) Inhibiting apoptosis by co-overexpressing *p35* with *Axn* partially suppressed the GSC loss. Student's *t*-tests were performed on the mean number of GSCs. *n* indicates the number of germaria counted for each experiment. Scale bars: 20 µm.

Previous studies have found that Wnt signaling in escort cells is essential for their function in promoting germline differentiation (Hamada-Kawaguchi et al., 2014; Kirilly et al., 2011; Luo et al., 2015; Mottier-Pavie et al., 2016; Upadhyay et al., 2016; Wang et al., 2015). Undifferentiated germ cells can be recognized by the presence of a spectrosome, a spherical organelle stained by anti-Hts; after germ cells differentiate into cysts, this organelle elongates and branches to become a fusome (Fig. 1A) (Lin et al., 1994). Consistent with these previous reports, we observed that inhibiting Wnt signaling in escort cells caused an accumulation of undifferentiated germ cells, identified by Hts-positive round spectrosomes (arrows in Fig. 2B-D, quantified in Fig. S2A).

Interestingly, in addition to this tumor-like germline, these germaria also exhibited a dramatic decrease in the number of GSCs (Fig. 2A-D, dashed circles). GSCs were identified by their attachment to the anterior cap cells and the presence of spectrosomes, which can appear either spherical or elongated during GSC divisions (de Cuevas and Spradling, 1998) (Fig. 2A). Control germaria had two or three GSCs located at the anterior tip. In contrast, by 7 days after *Axn* overexpression in escort cells, nearly all germaria contained fewer than two GSCs, with milder GSC-loss phenotypes observed with *sgg^S9A^* and *pan^DN^* (Fig. 2E). Although cap cells are known to control GSC number (Song et al., 2002; Xie and Spradling, 2000), their number, location and morphology were all unchanged (Fig. 1C-F,I). The severity of GSC loss was correlated with the severity of escort cell loss (compare Fig. 2E with Fig. 1H) but, interestingly, was inversely correlated with the severity of GSC differentiation defects (compare Fig. 2E with Fig. S2A). Thus, we hypothesized that escort cell death causes the loss of GSCs, as has been examined by one of us previously (Wang et al., 2011). To test this hypothesis, we co-expressed *p35*, an apoptosis inhibitor, with *Axn* in escort cells. *p35* partially rescued GSC loss, restoring the percentage of *Axn*-overexpressing germaria containing two or more GSCs from 0% to over 50% (Fig. 2F,G). These results indicate that Wnt signaling promotes escort cell survival to maintain GSCs. The inverse relationship between the extent of GSC loss and germline differentiation failure could explain why the GSC loss phenotype was not identified by previous laboratories investigating Wnt signaling in germline differentiation.

### Anterior escort cells are required for GSC maintenance

**Fig. 3.**
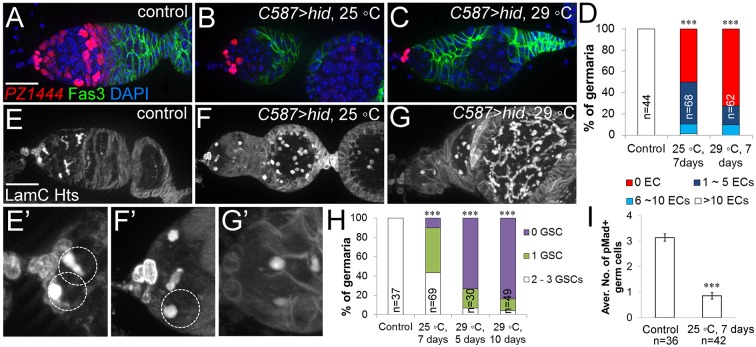
**Ablation of escort cells causes germline stem cell loss.** (A-C) Escort cell loss induced by moderate or high level of *hid* expression. Flies under the control of *C587Gal4* and *tubGal80^ts^* were switched upon eclosion from 18°C to 25°C to induce moderate expression of *hid* (B) or from 18°C to 29°C to induce high expression of *hid* (C). *PZ1444* (red) marked escort cells and cap cells. In C, the only *PZ1444*-labeled cells remaining were cap cells located at the anterior tip. (D) Quantification of escort cell loss in the conditions shown in A-C. (E-G) Removing escort cells from the germarium with *hid* resulted in loss of GSCs. (E′-G′) Magnified views of E-G showing GSCs (dashed circles), identified by anti-Hts staining and by their attachments to cap cells (labeled by anti-LamC). No GSCs are present in G. (H) Quantification of the number of GSCs in the conditions shown in E-G. (I) Quantification of the number of GSCs identified by pMad staining after *hid* expression in escort cells. ***P<0.001, Student's *t*-test was performed on the mean number of escort cells (D) or GSCs (H,I). *n* indicates the number of germaria counted for each experiment. Error bars indicate s.e.m. Scale bars: 20 µm.

To test directly whether escort cells are required for GSC maintenance, we ablated escort cells by forced ectopic expression of the pro-apoptotic gene *hid*. *hid* expression was restricted to escort cells in adults with *C587Gal4* and *tubGal80^ts^*. We chose two temperature-switch conditions to initiate *hid* expression: strong overexpression by switching from 18°C to 29°C, and moderate expression by switching from 18°C to an intermediate temperature 25°C (Fig. 3). Escort cell staining (*PZ1444*) confirmed the loss of escort cells induced by *hid* expression, with fewer escort cells remaining after high levels of *hid* expression (Fig. 3A-D). As expected, ablating escort cells resulted in an accumulation of undifferentiated germ cells (Fig. 3E-G, quantified in Fig. S2B), consistent with the function of escort cells as the differentiation niche (Kirilly et al., 2011). Importantly, ablating escort cells also caused a dramatic decrease in the number of GSCs (Fig. 3E-I). We unambiguously identified GSCs with pMad staining as well as by the presence of spectrosomes and attachment to cap cells (Fig. 3I, Fig. 5A,B). The severity of GSC loss was correlated with *hid* expression and escort cell loss: higher *hid* expression and fewer escort cells caused a more severe loss of GSCs (Fig. 3D,H).

**Fig. 4.**
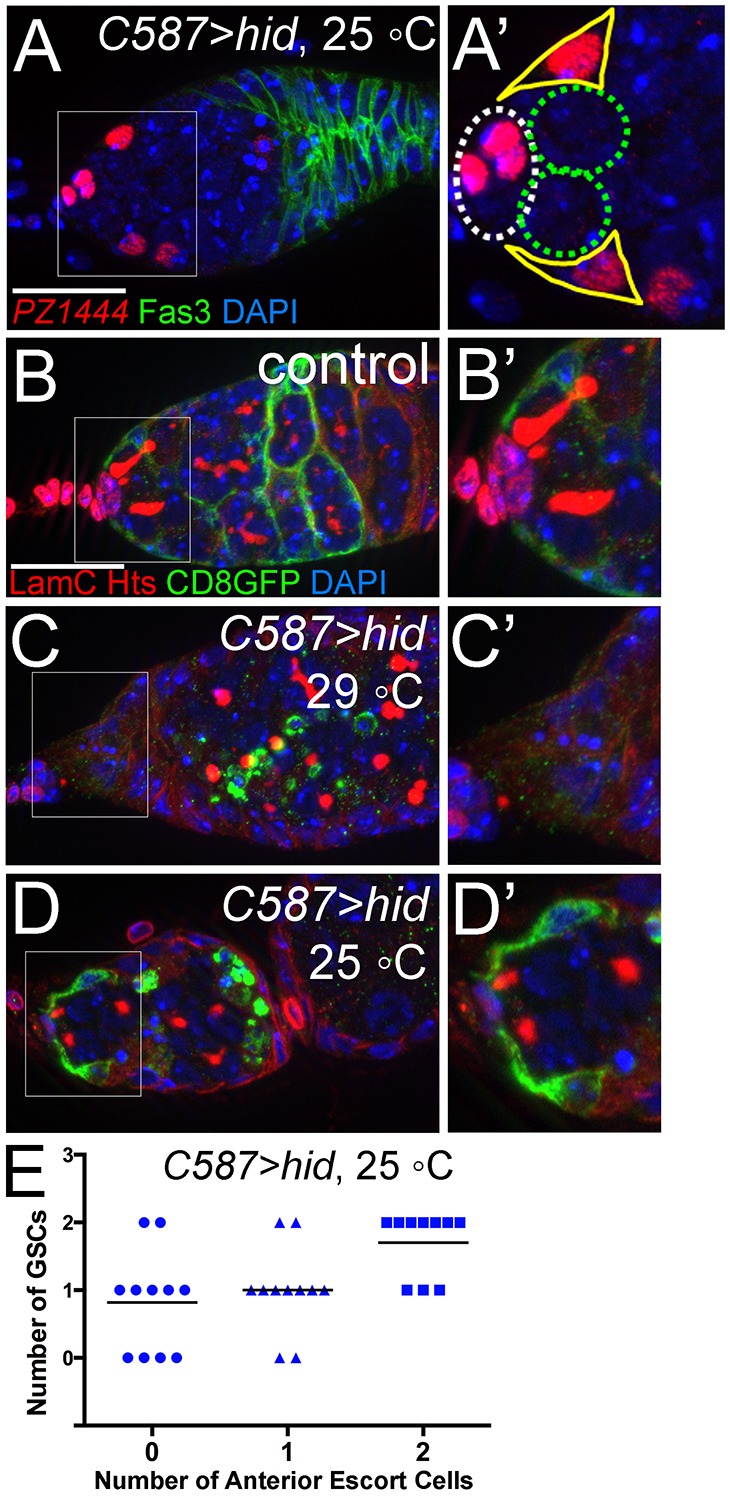
**Anterior escort cells are required for germline stem cell maintenance.** (A) Surviving escort cells remained in the anterior region of a germarium expressing moderate levels of *hid*. (A′) A magnified view of the boxed area in A showing the remaining anteriorly localized, GSC-contacting escort cells (outlined in yellow). White dashed circle delineates cap cells; green dashed circle, GSCs. (B-D) *Hid*-induced loss of anterior escort cells, visualized by plasma membrane-localized CD8GFP expressed with *C587Gal4*. Cap cells and GSCs were labeled with LamC and Hts, respectively (both red). (B′-D′) Magnified views of the boxed areas in B-D show the presence (B′,D′) or absence (C′) of anterior escort cells. (E) Quantification of the correlation between the number of anterior escort cells and GSCs in germaria expressing intermediate levels of *hid* (as shown in D). Mean values are shown as black lines. Scale bars: 20 µm.

We noticed that as escort cells die, the remaining escort cells clustered in the anterior of the germarium (Fig. 3B). Furthermore, germaria with at least two anteriorly localized, GSC-contacting escort cells remaining usually had both of their GSCs present (Fig. 4A,D,E). We use the term ‘anterior escort cells’ to refer to the most anteriorly located escort cells that encase GSCs with their cytoplasmic processes (Fig. 4B,D). To clearly outline and identify each escort cell, a membrane-localized GFP (mCD8GFP) was expressed in escort cells with *C587Gal4*. With this tool, we observed that the presence of anterior escort cells was correlated with the presence of GSCs (Fig. 4B-D, quantified in Fig. 4E). These results indicate that anterior escort cells are an integral component of the GSC niche.

### Anterior escort cells promote BMP signaling in GSCs and GSC anchorage within the niche

**Fig. 5.**
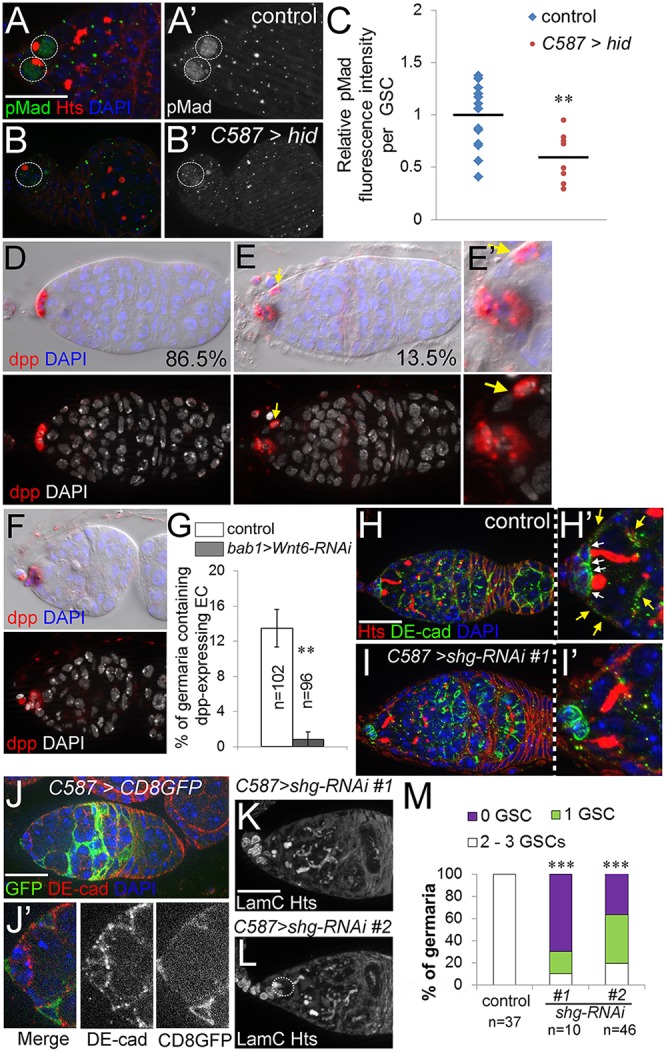
**Anterior escort cells promote both BMP signaling in GSCs and GSC adhesion within the niche.** (A-B′) Ablating escort cells by overexpressing *hid* resulted in loss of BMP signaling in GSCs (circled). pMad staining was used as a marker for BMP signaling. (C) Quantification of the relative intensity of pMad staining in GSCs residing in the niche; mean fluorescence is indicated by the line. (D-E′) RNAscope *in situ* hybridization against *dpp* (red) reveals that although most control germaria express *dpp* only in cap cells, about 13% of control germaria express *dpp* in an anterior escort cell (arrows in E,E′). RNAscope generates a fluorescent signal (lower panels) and a chromogenic deposit visible by differential interference contrast (top panels, black). (F) The transient RNAscope *dpp* signal is not present in anterior escort cells when *Wnt6* is knocked down in cap cells. (G) Quantification of germaria in the conditions shown in D-F. Flies contained *tubGal80^t^*^s^ and were switched upon eclosion from 18°C to 29°C for 8-10 days before dissection, to restrict *Gal4* expression to adult cap cells. (H,H′) DE-cadherin (green) is expressed at the interface of the two anterior-most escort cells and GSCs (yellow arrows), in addition to the junction between cap cells and GSCs (white arrows). (I,I′) Knocking down *shg*, encoding DE-cadherin, from escort cells resulted in loss of GSCs from the niche. Magnified image (I′) shows two GSCs detaching from the cap cells. (J,J′) Colocalization of DE-cadherin with membrane-bound CD8-GFP only on escort cells (driven by *C587Gal4*) unambiguously identifies DE-cadherin between GSCs and escort cells. (K,L) Two independent *shg* RNAi constructs expressed in escort cells caused loss of GSCs (circled). The germarium in K contains no GSCs. (M) Quantification of GSC number in the conditions shown in K,L. **P<0.01, ***P<0.001 (Student's *t*-test). *n* indicates the number of germaria counted for each condition. Error bars represent s.e.m. Scale bars: 20 µm.

To identify mechanisms underlying the requirement for anterior escort cells in the GSC niche, we examined BMP signaling in those *hid*-expressing germaria that still retained one or two GSCs, using phosphorylated Mad (pMad) as an indicator of BMP signaling activity. In control germaria, niche-derived BMP signaling is restricted to GSCs and is required for repressing differentiation (Fig. 5A; Chen and McKearin, 2003; Song et al., 2004). *hid* expression in escort cells caused a significant decrease of pMad levels in the remaining GSCs (Fig. 5A-C), in addition to causing GSC loss, suggesting that escort cells are directly required for maintaining BMP signaling activity in the GSCs.

To determine if anterior escort cells could be directly signaling to GSCs via the BMP pathway, we performed whole-mount *in situ* hybridization against the BMP ligand *dpp*. As traditional *in situ* hybridizations are difficult in the germarium, we adapted RNAscope technology for use in *Drosophila* tissues, as it offers reduced background and highly amplified signal (Player et al., 2001; Wang et al., 2012). Cap cells, known to express high levels of *dpp*, served as a positive control (Fig. 5D). For a negative control, we knocked down *dpp* in cap cells with two different RNAi lines and observed the RNAscope *dpp* signal to be reduced or absent in cap cells with both lines (Fig. S3). Importantly, in each of three experiments about 13% of control germaria had a clear *dpp* signal in a single anterior escort cell (Fig. 5E,E′,G), consistent with previous observations (Liu et al., 2010; Wang et al., 2008). Although the placement of the *dpp*-expressing escort cell was always within Region 1, in 2/13 cases it was at the posterior border of Region 1 and not adjacent to the cap cells in the anterior. Because the *dpp* RNAscope signal in anterior escort cells was either strong or absent, in a binary fashion, we do not interpret the incomplete penetrance to mean that *dpp* ligand hovered near the threshold of detection (as observed in cap cell knockdown controls, Fig. S3). Rather, these results indicate that anterior escort cells transiently express *dpp*, probably in response to events in the GSC niche. Notably, *dpp* signals were not observed in escort cells outside of Region 1 (*n*=102).

We next investigated the distribution of DE-cadherin (Shg in *Drosophila*), which anchors GSCs to the cap-cell niche and is localized to the junctions formed between cap cells and GSCs (Song et al., 2002). In addition to this previously reported pattern, we observed DE-cadherin at the interface of GSCs and anterior escort cells (Fig. 5H,H′, yellow arrows), most clearly shown by the colocalization of DE-cadherin and *C587*-driven CD8-GFP on escort cell membranes (Fig. 5J,J′). To test its function, we knocked down the gene encoding DE-cadherin only in escort cells using *C587Gal4*. Escort cell knockdown of DE-cadherin with either of two different RNAi lines caused a significant decrease in the number of GSCs (Fig. 5I,K-M). In Fig. 5I′, two GSCs are shown detaching from the cap cells as revealed by their elongated spectrosome morphology, in a germarium with escort cell knockdown of DE-cadherin that still has normal levels of DE-cadherin at the cap cells. Thus, the anterior escort cells contribute to anchoring GSCs within the niche by expressing DE-cadherin in addition to promoting full-strength BMP signaling in GSCs.

### Cap cells secrete Wnt6 to maintain GSCs

To investigate how Wnt signaling contributes to GSC maintenance, we considered the seven Wnt ligands in the *Drosophila* genome, four of which have been shown to be expressed in the germarium by previous *in situ* hybridization studies (Luo et al., 2015). Among these, *wingless* (*wg*) is expressed in cap cells and is required for follicle stem cell proliferation (Sahai-Hernandez and Nystul, 2013; Song and Xie, 2003; Wang and Page-McCaw, 2014). However, neither the *wg* temperature-sensitive mutant (Song and Xie, 2003) nor *wg* RNAi (Wang and Page-McCaw, 2014) exhibited GSC loss (not shown). *Wnt2* and *Wnt4* are expressed in escort cells, and it has been reported that loss of *Wnt4* from escort cells resulted in accumulation of undifferentiated germ cells (Hamada-Kawaguchi et al., 2014; Mottier-Pavie et al., 2016; Upadhyay et al., 2016), a phenotype enhanced by knocking down *Wnt2* simultaneously from escort cells (Wang et al., 2015). When we mutated or knocked down *Wnt4* in escort cells, we found that germaria lost GSCs, in addition to accumulating undifferentiated germ cells (Fig. S4). *Wnt*2 whole-animal mutants also showed a mild GSC-loss phenotype, although when *Wnt2* was knocked down in escort cells, GSCs were unaffected (Fig. S4). Thus Wnt4 and possibly Wnt2 signal in an autocrine manner to promote GSC maintenance.

**Fig. 6.**
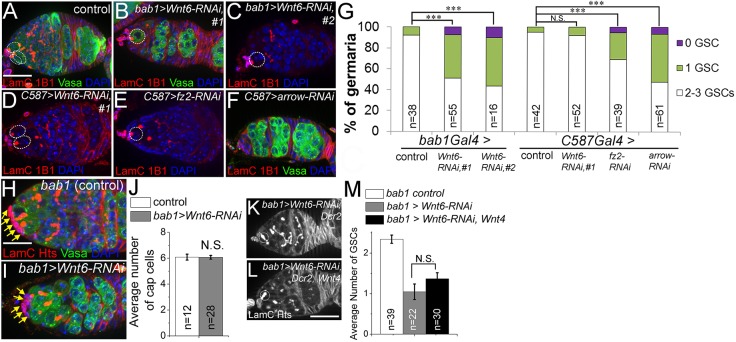
**Wnt6 from the cap cells is required for GSC maintenance.** (A-C) *Wnt6* knockdown driven by the cap cell-specific driver *bab1-Gal4* using two different *Wnt6* RNAi lines resulted in GSC loss. (D) In contrast to cap cell expression, *Wnt6* RNAi in escort cells using *C587Gal4* did not cause significant GSC loss. (E,F) Knocking down the Wnt receptor *fz2* or co-receptor *arrow* from escort cells resulted in GSC loss. (G) Quantification of GSC number in the conditions shown in A-F. (H,I) Knocking down *Wnt6* in cap cells did not affect the number of cap cells. LamC labeled the nuclear envelope of cap cells, indicated by yellow arrows. (J) Quantification of cap cell number in the conditions shown in H,I. (K,L) Overexpressing *Wnt4* did not suppress the GSC loss caused by knocking down *Wnt6* from the cap cells. (M) Quantification of GSC number in the conditions shown in K,L. All flies contained *tubGal80^t^*^s^ and were switched upon eclosion from 18°C to 29°C for 10 days (A-C) or 12-13 days (D-F) before dissection, to allow ectopic gene expression in escort cells only in the adult stage. N.S., not significant; ***P<0.001 (Student's *t*-test). In A-F,K,L, GSCs are indicated by dotted white circles. *n* indicates the number of germaria counted for each condition. Error bars represent s.e.m. Scale bars: 20 µm.

*Wnt6* is specifically expressed in cap cells in the germarium (Luo et al., 2015). Cap cell-specific knockdown of *Wnt6* throughout development results in the accumulation of undifferentiated germ cells (Luo et al., 2015), a phenotype we confirmed in whole-animal *Wnt6* knockout mutants (not shown). To explore the possibility of a role for *Wnt6* in maintaining the GSC niche in adults, we performed adult-onset knockdown of *Wnt6* in cap cells with *bab1Gal4*, or in escort cells with *C587Gal4*, each with *Gal80^ts^* (Fig. 6). When *Wnt6* was knocked down in cap cells, significant GSC loss occurred without accumulation of undifferentiated germ cells, a phenotype confirmed using two independent *Wnt6* RNAi lines (Fig. 6A-C,G). In contrast, knocking down *Wnt6* from escort cells did not cause significant GSC loss (Fig. 6D,G). Thus, cap cells, but not escort cells, express *Wnt6* to regulate GSC maintenance. Although *Wnt6* is necessary for maintaining GSC number, it is not sufficient to produce excess GSCs: overexpression of *Wnt6* in cap cells or escort cells, or overexpression of its downstream signal transducer *arm^S10^* in escort cells, did not alter GSC numbers or germline differentiation (Fig. S5). Importantly, *Wnt6* knockdown in cap cells did not affect the cap cells themselves: the number of cap cells (Fig. 6H-J) and the level of DE-cadherin in cap cells (Fig. S6) remained unchanged, indicating that *Wnt6* does not maintain GSCs in an autocrine manner, via adherens junctions between cap cells and GSCs.

Importantly, when *Wnt6* was knocked down in cap cells, the transcription of *dpp* was lost specifically in the anterior escort cells (Fig. 5F,G) and reduced pMad staining was observed in GSCs (Fig. S7), indicating a reduction in BMP signaling within these GSCs. In contrast, the level of DE-cadherin between GSCs and anterior escort cells was unchanged when *Wnt6* was knocked down in cap cells (not shown), and overexpression of DE-cadherin in escort cells did not suppress GSC loss in *Wnt6^KO^* mutants (Fig. S8).

Because Wnt6 acts in a paracrine manner, we identified the possible receptor and co-receptor for Wnt6 in escort cells. We performed escort-cell specific knock down of *frizzled* (*fz*) with three different RNAi lines, *frizzled2* (*fz2*) with two different RNAi lines, or *arrow*; both *fz2* and *arrow* (Fig. 6E-G), but not *fz* (data not shown), are required in escort cells to promote GSC maintenance. To test whether *Wnt4* and *Wnt6* can function interchangeably to promote GSC maintenance, we forced the expression of *Wnt4* in cap cells that had *Wnt6* knocked down. As shown in Fig. 6K-M, forced expression of *Wnt4* in cap cells did not suppress the GSC-loss phenotype caused by *Wnt6* RNAi in cap cells, suggesting that Wnt4 and Wnt6 either require different partners or activate distinct pathways in escort cells. Thus, Wnt6 is an important paracrine signal expressed in cap cells and acting on escort cells to regulate GSC maintenance.

### Cap cells promote the survival of anterior escort cells via Wnt6

**Fig. 7.**
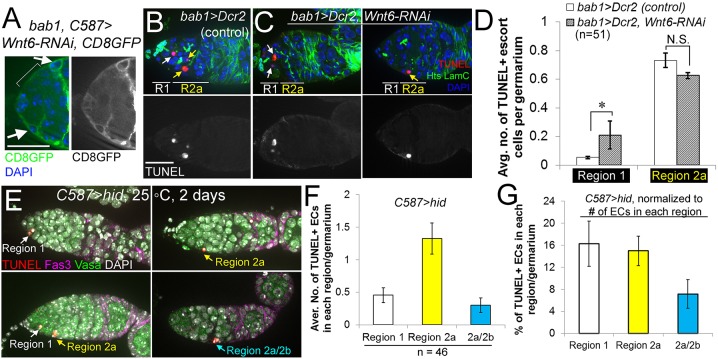
**Wnt6 promotes the survival of anteriorly localized escort cells.** (A) Knockdown of *Wnt6* from cap cells resulted in escort cell vacancies in the GSC niche. CD8-GFP labeling of escort-cell membranes (arrows) reveals an area devoid of escort cells (bracket). Compare with control germaria in Fig. 4B and Fig. 5J, or with *C587>hid* germaria in Fig. 4D. (B-E) TUNEL (red) detects apoptotic escort cells. TUNEL^+^ escort cells are indicated by white arrows in Region 1, yellow arrows in Region 2a, and blue arrows at the boundary of Region 2a/2b. (C) *Wnt6* RNAi induced apoptosis in escort cells located in Region 1, but not Region 2a of the germarium. (D) Quantification of the average number of TUNEL^+^ escort cells per germarium, in the conditions shown in B,C. (E-G) In contrast, apoptotic cells are spread throughout the germarium when *C587>hid* killed escort cells. For each region of the germarium, raw numbers of TUNEL^+^ escort cells in *C587>hid* are quantified in F and the percentage of TUNEL^+^ escort cells is quantified in G. Flies were under the control of *C587Gal4* and *tubGal80^ts^* and were switched upon eclosion from 18°C to 25°C to induce moderate expression of *hid*. *n* indicates the number of germaria counted for each condition. N.S., not significant; *P<0.05 (Student's *t*-test). Error bars represent s.e.m. Scale bars: 20 µm.

Interestingly, when *Wnt6* was knocked down in cap cells, we observed vacancies in the anterior escort cell territory not seen in controls, most clearly visualized by expressing membrane-anchored CD8GFP in cap and escort cells (compare Fig. 7A with Fig. 4B′ and Fig. 5J′). Anterior vacancies were not observed under other conditions, even when escort cell death was caused by overexpressing *hid*; in this case, escort cells were observed instead to cluster toward the GSC niche (Fig. 3B and Fig. 4A,D). We hypothesized that cap cells express *Wnt6* to promote anterior escort cell survival, thus regulating GSC number. To test this hypothesis, we examined the distribution of apoptosis in control and *Wnt6* knockdown germaria. In control germaria, TUNEL-labeled apoptotic cells were occasionally found in Region 1 or Region 2a (Fig. 7B,D). In contrast, *Wnt6* RNAi in cap cells caused a significant increase in apoptotic cells in Region 1, the site of anterior escort cells, whereas Region 2a remained unchanged (Fig. 7C,D). These results indicate that Wnt6 preferentially regulates the survival of anterior escort cells located close to its cap-cell source.

Because anterior escort cells and GSCs were maintained even when escort cells were actively killed by *hid* expression, we were curious about the spatial distribution of cell death in these *hid*-expressing germaria in which Wnt6 signaling is intact (Fig. 7E). We found that escort cell death rates increased proportionally across the germarium when *hid* was expressed with *c587-Gal4* (compare Fig. 7D with 7F). Because more *c587*-expressing cells are found in Region 2a than in Region 1, we normalized the death rate to cell number and found that death occurred at equal frequency in Regions 1 and 2a (Fig. 7G). These results indicate that even though cell death was distributed across the germarium, when Wnt6 signaling was intact surviving ECs clustered toward the anterior of the germarium, taking up positions in the GSC niche. Thus, the Wnt6 survival signal appears to be important for maintaining the spatial organization of escort cells in the niche and for replacing them when they die.

### Anterior escort cells might be derived from posterior cells

**Fig. 8.**
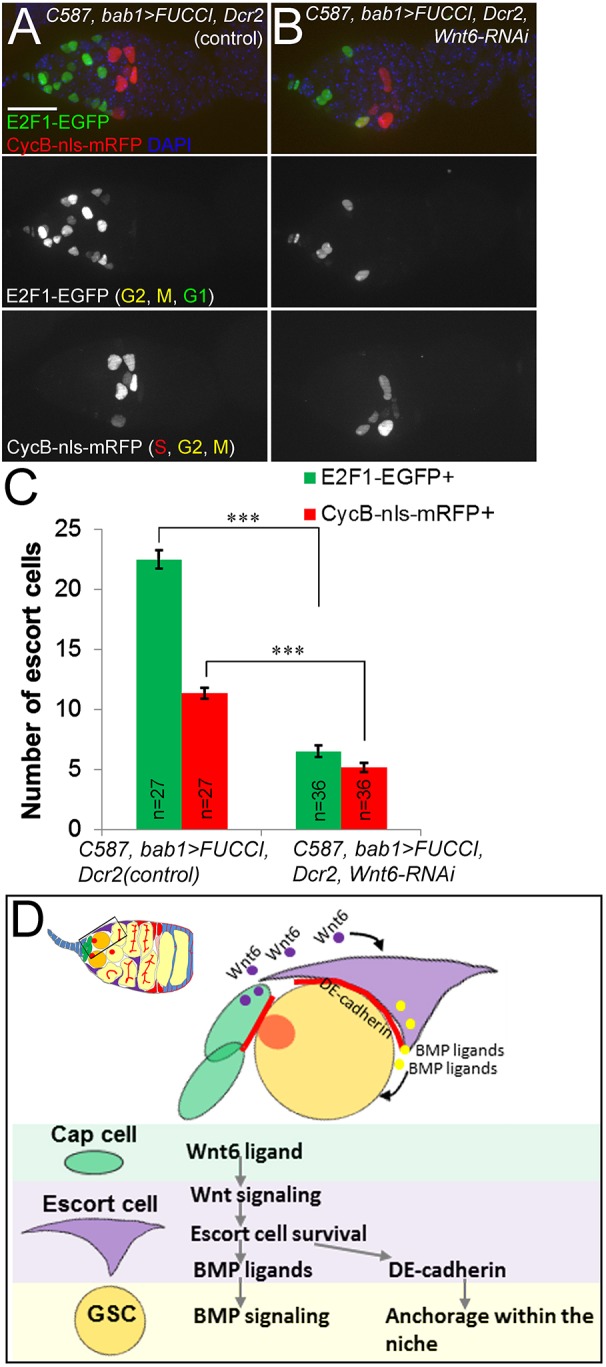
**Wnt6 maintains anterior escort cells to promote GSC survival.** (A) *C587Gal4* expression of the cell-cycle indicator FUCCI identified two distinct cell populations: the anterior region contained exclusively E2F1-EGFP-positive green cells that are quiescent in G1 phase, whereas the posterior regions contained cycling cells expressing CycB-nls-mRFP (red in S phase, or yellow in M/G2 phases). (B) *Wnt6* RNAi significantly reduced the number of both cell populations. (C) Quantification of E2F1-EGFP-positive and CycB-nls-mRFP-positive escort cells as shown in A,B. ***P<0.001 (Student's *t*-test). Error bars represent s.e.m. (D) Model for how cap cells organize the germline stem cell niche in the fly germarium. Cap cells secrete Wnt6 ligands that act on abutting anterior escort cells to activate Wnt signaling, which is essential for their survival. Anterior escort cells function as an integral component of the GSC niche by promoting BMP signaling in GSCs and DE-cadherin-mediated anchorage of GSCs within the niche.

To gain insight into how anterior escort cells are replaced around the niche, we examined the cell cycle status of all escort cells by expressing *Drosophila* FUCCI (fluorescence ubiquitination-based cell cycle indicator; Zielke et al., 2014) with *C587Gal4*. With this system, nuclei in G1 phase are labeled green, S phase red, and G2 and M phases yellow (red+green). In control germaria, two distinct populations were evident: cells located in Region 1 and the anterior part of Region 2a were exclusively labeled green, indicating a quiescent G1 phase, whereas cells located in the posterior part of Region 2a and at the 2a/2b boundary were labeled red, yellow or green, indicating active cycling (Fig. 8A). During the course of our analysis, the posterior *C587*-expressing cells were re-classified as stem cells, which give rise to escort cells or follicle cells depending in part on their level of Wnt signaling (Reilein et al., 2017). Our FUCCI data are consistent with these new findings. Interestingly, *Wnt6* knockdown decreased the number of cells in both populations, cycling and quiescent, albeit to a lesser degree in the cycling posterior group, indicating that *Wnt6* is required for maintaining escort cell number (Fig. 8A-C).

The observation that cells divide exclusively in the posterior half of the germarium suggests that occasional vacancies caused by stochastic cell death are filled by anterior movement of escort cells in a *Wnt6*-dependent manner. Consistent with this model, we observed that bromodeoxyuridine (BrdU)-labeled escort cells were identified in the anterior region of germaria after many days of *in vivo* chase (Fig. S9), supporting anterior movement. Such anterior migrations of escort cells have been documented by Reilein et al. (2017), who captured the anterior migration of labeled escort cells by live imaging of germaria cultured *ex vivo*. Thus, escort cells move anteriorly, filling any available spots in the germline stem cell niche. Our data show that Wnt6 is required for survival of anterior escort cells in the GSC niche, and it suggests that *Wnt6* might facilitate replacement of those cells after their stochastic death. Together, these data support a model in which cap cells secrete Wnt6 to maintain anterior escort cells in the GSC niche, and these escort cells function with the cap cells to anchor GSCs physically with DE-cadherin and maintain GSC stemness by producing Dpp ligands (Fig. 8D).

## DISCUSSION

### Anterior escort cells are a crucial component of the GSC niche

Previously, it was held that the GSC niche was composed of cap cells located at the anterior tip of the germaria. Cap cells produce BMP ligands to inhibit differentiation, and they anchor GSCs via DE-cadherin-mediated adherens junctions for continuous self-renewal (Chen and McKearin, 2003; Song et al., 2002, 2004; Xie and Spradling, 2000). In this study, we demonstrate that, in addition to cap cells, the anterior-most escort cells are required to maintain GSCs in the niche. Although these anterior escort cells have not been identified with a specific cell marker, multiple lines of evidence point to anterior escort cells having a crucial niche function. First, like cap cells, anterior escort cells form adherens junctions with GSCs via DE-cadherin, and when DE-cadherin is knocked down in all escort cells, GSCs are lost; this requirement suggests that anterior escort cells participate with cap cells in physically attaching GSCs in the niche. Second, when all escort cells are challenged and dying, as a result of either impaired Wnt signaling or direct killing with *hid*, remaining escort cells cluster in the anterior around the GSCs. GSC loss is evident only after nearly all escort cells have died, leaving visible anterior vacancies around the GSCs. Third, when all escort cells are dying, GSCs lose the full-strength BMP signaling that is necessary to maintain the stem-cell state; in control germaria, the BMP ligand Dpp is expressed exclusively in escort cells of Region 1, primarily in the anterior-most escort cells, in an apparently transient manner. Fourth, Wnt6 ligand is required specifically in cap cells and not in escort cells for maintaining anterior escort cell survival, for maintaining anterior escort cell architecture within the niche, for full-strength BMP signaling in GSCs, and for maintaining GSCs in the niche. Together, these data demonstrate that anterior escort cells are crucial components of the GSC niche. Furthermore, anterior escort cells share the niche hallmarks of *dpp* expression and DE-cadherin attachments to GSCs, both of which are required in escort cells as well as cap cells for GSC maintenance in the niche.

This model of escort cell participation in the GSC niche is consistent with and extends some previous observations. One of us (Wang et al., 2011) previously showed that when escort cells were knocked down for the histone modifier *eggless*, escort cells slowly died with a concomitant loss of GSCs, but this phenotype was not quantified or further investigated. Several labs have shown by RT-PCR (Rojas-Ríos et al., 2012; Song et al., 2004) or by a conventional and challenging *in situ* hybridization method (Liu et al., 2010; Wang et al., 2008) that escort cells contribute Dpp ligand to the germarium environment. Importantly, when *dpp* was knocked down in all escort cells with adult-specific expression of *ptc-Gal4*, GSC loss was observed (Rojas-Ríos et al., 2012). These results are all consistent with our data and model of anterior escort cell function.

### Two populations of escort cells: pro-stem and pro-differentiation

Escort cells are better known as the ‘differentiation niche’, because they are required for the proper differentiation of GSC progeny. Indeed, several studies have shown that escort cells, and specifically Wnt signaling in escort cells, are essential for germline differentiation (Hamada-Kawaguchi et al., 2014; Luo et al., 2015; Mottier-Pavie et al., 2016; Upadhyay et al., 2016; Wang et al., 2015). Like these groups, we observed a germline differentiation phenotype when Wnt signaling was compromised in escort cells in addition to the GSC-loss phenotype, but, interestingly, the two phenotypes were inversely correlated: manipulations that resulted in the greatest number of undifferentiated germ cells (such as *sgg^S9A^* overexpression or moderate induction of *hid*) were those that maintained a moderate escort cell number, and these displayed the lowest level of GSC loss; reciprocally, manipulations that resulted in the greatest loss of GSC (such as Axn overexpression or high induction of *hid*) were those that induced a severe loss of escort cells, and these displayed the lowest levels of undifferentiated germ cells. We conclude that the earliest phenotype caused by escort cell death is a failure of germline differentiation, appearing as a germline tumor. The loss of GSCs from the niche is a later phenotype, appearing only after nearly all the escort cells have been lost from the germarium, which happens when Wnt signaling is strongly impaired or when *hid* is highly expressed. The inverse correlation makes sense because when GSCs are lost from the niche, fewer of their cystoblast progeny are born to populate a germline tumor. We expect that studies analyzing the role of Wnt in germ cell differentiation might not have detected the weak loss of GSCs in their strong differentiation mutants, and further, weak GSC loss is hard to detect in the presence of many undifferentiated germ cells because of the large number of spectrosomes. These two phenotypes represent two distinct functions of escort cells: promoting germline stemness in the GSC niche at the anterior of the germarium, and promoting germline differentiation in the differentiation niche in more posterior positions. Both Wnt6 and Hh, signaling from cap cells to anterior escort cells, are positioned appropriately to signal this switch in escort cell function (Luo et al., 2015; Rojas-Ríos et al., 2012; this study).

### Wnt6 as an anterior escort cell maintenance signal

Intriguingly, we find that cap cells signal via Wnt6 to anterior escort cells to promote their survival. This signaling between two different niche cell types is crucial for niche function, as without Wnt6, niche escort cells die, *dpp* expression in anterior escort cells is lost, BMP signaling in GSCs is decreased, and GSCs are lost. It seems likely that the loss of *dpp* expression is an indirect effect of losing the anterior escort cells themselves, rather than a direct effect of the loss of Wnt signaling, as it has been reported that cap cell-derived Wnt ligands limit rather than promote *dpp* signaling (Luo et al., 2015). Also, it has been previously shown that cap cell-derived Hh ligands promote *dpp* expression in escort cells (Rojas-Ríos et al., 2012). Thus, we favor a model in which Wnt6 is important for anterior escort cell survival and recruitment. In support of this model, we observed that in the presence of intact Wnt6 signaling, when escort cells were killed by *hid*, surviving escort cells routinely clustered at the GSC niche, even though escort cell death occurred evenly across the germarium. Indeed, GSCs were maintained in the niche until virtually all escort cells had died, when there were few or no remaining escort cells to fill vacancies in the niche. Escort cells behaved very differently, however, when *Wnt6* was knocked down in cap cells. Without *Wnt6*, we observed an increase in cell death specifically in the anterior of the germarium, and lost cells were not replaced, leaving functional vacancies in the GSC niche. Thus, cap cell-produced Wnt6 seems to ensure continued occupancy of escort cells in the GSC niche. It is also possible that Wnt6 could coordinate the niche cell types during changes in niche size, as previous studies have shown that both the numbers of GSCs and cap cells decrease in response to a poor diet and increase under rich food conditions (Bonfini et al., 2015; Hsu and Drummond-Barbosa, 2009).

Anterior escort cell replacements appear to derive from the more posterior cycling somatic cells, labeled by FUCCI. Based on recent work by Reilein et al. (2017), it appears that these cycling cells are stem cells from which both follicle and escort cells derive. The anterior migration of stem cell daughters into escort cell territory has been captured by live imaging *ex vivo* (Reilein et al., 2017), strong evidence that anterior movement occurs also *in vivo*. Furthermore, we observed some BrdU-labeled cells that probably migrated from this cycling area into Region 1. Thus, Wnt6 might act as a homing signal for newly born escort cells, attracting them to the anterior-most location in the GSC niche.

### Do escort cells and cap cells relay distinct types of information to the GSCs?

It has been proposed that a stem cell niche acts as an ‘interlocutor’ or interpreter, relaying information about the status of the organism or tissue to the stem cells. Because of this interpreter role, it is expected that niches would be composed of multiple cell types to report different types of information (Scadden, 2014). Indeed, some mammalian somatic stem cell niches are known to be composed of multiple cell types. The bone marrow niche for hematopoietic stem cells (HSCs), one of the best understood mammalian stem cell niches, is composed of multiple cell types, including different endothelial cells in the circulatory system and cells in the nervous and immune systems (Acar et al., 2015; Birbrair and Frenette, 2016; Kirkeby et al., 2016). Another example is the mammalian intestinal stem cell niche, composed of paneth cells, pericryptic fibroblasts and smooth muscle cells (Rezza et al., 2014). In this study, we demonstrate that escort cells are an essential and non-redundant niche cell type, acting in concert with the cap cells to form the *Drosophila* ovarian GSC niche. Following the interlocutor model, what could each of these two cell types be communicating to the GSCs? Germline differentiation and the development of gametes need to be coordinated with at least two types of information: nutritional status of the organism, and the level of threat to the genome from transposable elements. The cap cells are known to gather information on the nutritional status of the organism, as they change their number or alter the availability of signaling ligands in response to diet (Bonfini et al., 2015; Hartman et al., 2013; Hsu and Drummond-Barbosa, 2009). Interestingly, a recent study has shown that escort cells respond to transposable element activation by downregulating Wnt4 levels, a potentially direct mechanism by which escort cells communicate the level of transposon threat to the germline (Upadhyay et al., 2016). In this scenario, increased transposon activity leads to reduced Wnt4 signaling, and our data shows that reduced Wnt4 results in potentially corrupted GSCs being lost from the perpetuity of the niche. Thus, both cap cells and escort cells are poised to transmit crucial information relevant to gamete development through the GSC niche.

## MATERIALS AND METHODS

### Fly stocks and maintenance

Flies were cultured on cornmeal-molasses media at 25°C unless otherwise noted. Age-matched females were mated with wild-type males and were fed with fresh wet yeast that was changed every other day until dissection. For adult-onset gene expression using the *Gal4/Gal80^ts^* system, flies were raised at 18°C, shifted 1-2 days after eclosion to 25°C or 29°C and aged 7-10 days before dissection.

The following stocks are described in FlyBase and were obtained from Bloomington *Drosophila* Stock Center: *UAS-Axn.GFP* (#7224), *UAS-arm^S10^* (#4782), *UAS-pan^DN^* (*UAS-pan.dTCFΔN*) (#4785), *UAS-p35* (#5072), *UAS-mCD8GFP* (#5137), *bab1Gal4^Agal4-5^* (#6802), *bab1Gal4* (FBal0242651, gift from Acaimo Gonzalez-Reyes) (Bolívar et al., 2006), *tubGal80^ts^* (#7017), *Wnt4^EMS23^* (#6150), *Wnt4^C1^* (#6151), *Wnt2^L^* (#6909), *Wnt2^O^* (#6958), *UASp-shg.GFP* (#58445), Fly-FUCCI (UAS-GFP.E2f1.1-230, UAS-mRFP1.NLS.CycB.1-266) (#55121). Other lines include *C587-Gal4* (a gift from Daniela Drummond-Barbosa, Johns Hopkins University, Baltimore, MD, USA), *PZ1444* (a gift from Allan Spradling, Carnegie Institution for Science, Baltimore, MD, USA), *fz3-RFP* (Olson et al., 2011), *UAS-hid* (a gift from Julien Royet, IBDM, Marseille, France), *UAS-Wnt4* (a gift from Nicholas Tolwinski) (Peradziryi et al., 2011), *Wnt6^KO^* (*Wnt6* knockout generated by homologous recombination-based targeting, gift from Aurelio Teleman; Doumpas et al., 2013), *UAS-sgg^S9A^* (*Drosophila* Genomics and Genetic Resources, Kyoto, Japan). RNAi lines used include *Wnt6-RNAi*, #1 (Vienna *Drosophila* RNAi Center, VDRC, #104020), *Wnt6-RNAi*, #2 (Vienna *Drosophila* RNAi Center, #27610), *Wnt2-RNAi* (Transgenic RNAi project, TRiP, HMS02826), *Wnt4-RNAi* (VDRC, #104671), *shg-RNAi*, #1 (TRiP, HMS00693), *shg-RNAi*, #2 (TRiP, JF02769), *fz2-RNAi* (TRiP, JF01378 and JF02722), *arrow*-*RNAi* (VDRC, #6707), *fz-RNAi* (TRiP, HMS01308, JF01258 and JF01481), *dpp-RNAi* #1 (TRiP JF01371), and *dpp-RNAi* #2 (TRiP HMS00011).

### Immunohistochemistry

Ovaries were stained according to previously described protocols (Wang and Page-McCaw, 2014). Briefly, dissected ovaries were fixed in 4% paraformaldehyde (Ted Pella) for 18 min, washed thoroughly with PBST (PBS with 0.1% Triton X-100), blocked in PBST containing 5% normal goat serum, and then incubated overnight with primary antibodies diluted in blocking solution at 4°C. The next day, ovaries were washed in PBST for 2 h, incubated with secondary antibodies at room temperature for 3-4 h, and washed in PBST for another 2 h. Stained samples were mounted in Vectashield containing DAPI (Vector Laboratories). Primary antibodies from the Developmental Studies Hybridoma Bank (DSHB) were as follows: mouse anti-Fas3 (7G10, 1:8), mouse anti-Hts (1B1, 1:5), mouse anti-β-galactosidase (40-1a, 1:50), mouse anti-LamC (LC28.26, 1:20), rat anti-Vasa (1:10) and rat anti-DE-cadherin (DCAD2, 1:7). Other primary antibodies used were: mouse anti-GFP (clone N86/38, UC Davis/NIH NeuroMab Facility, 1:5) and rabbit anti-Smad3 (phospho S423+S425, Epitomics, Cat# 1880-1, 1:200). Secondary antibodies used were Cy3-conjugated and FITC-conjugated goat anti-mouse IgG1, Cy3-conjugated and FITC-conjugated goat anti-mouse IgG2a (all from Jackson ImmunoResearch, used at 1:500), goat anti-rabbit IgG and goat anti-rat IgG conjugated to Alexa Fluor 488 (Molecular Probes, used at 1:500). Because antibodies were used to label cells types and tissues, they were validated by examining staining in wild-type animals and comparing to known patterns.

### TUNEL staining

Ovaries were dissected in Schneider's *Drosophila* medium (Gibco), fixed in 4% paraformaldehyde (Ted Pella) in PBS, washed thoroughly in PBS, and permeabilized with PBS containing 0.1% Triton X-100 and 0.1% sodium citrate. One-hundred microliters of the TUNEL reaction mixture (*In Situ* Cell Death Detection Kit TMR Red, Roche) was added to five pairs of ovaries, and samples were incubated at 37°C in the dark for 1 h. Then ovaries were washed thoroughly in PBST, blocked and co-stained with primary antibodies overnight as described above.

### RNAscope assay

We devised a protocol for RNAscope based on methods for whole-mount zebrafish staining (Gross-Thebing et al., 2014). Briefly, *Drosophila dpp* probes were designed and made to order by Advanced Cell Diagnostics to target 682-1673 of NM_057963.5 (accession number from NCBI). RNAscope was performed on whole-mount ovaries in an Eppendorf tube. About ten pairs of ovaries were dissected into ovarioles in *Drosophila* Schneider's Medium, washed once with PBS and fixed in 4% paraformaldehyde in PBS overnight. Ovaries were washed 3×5 min in PBT (PBS containing 0.1% Tween-20) and dehydrated in a series of 25%, 50%, 75% and 100% methanol in PBT. Following the last wash, ovaries were stored in 100% methanol at −20°C for at least 2 h. Then methanol was removed completely, and ovaries were air-dried at room temperature for 30 min. Protease digestion using Pretreat 3 (RNAscope H_2_O_2_ & Protease Plus Reagents; ACD, 322330) was performed on ovaries at room temperature for 5 min followed by rinsing in PBT three times. *dpp* probe hybridization was performed overnight at 40°C in ACD HybEZ Hybridization System (110VAC) (ACD, 321461). The following day, ovaries were washed in RNAscope wash buffer (ACD, 310091) for 3×5 min, re-fixed in 4% paraformaldehyde in PBS at room temperature for 10 min and washed for 3×5 min. Ovaries were then incubated with a series of amplifier solutions (Amps) contained in RNAscope 2.5 HD Detection Reagent – RED (ACD, 322360) according to the manufacturer's instructions. Briefly, ovaries were incubated in Amp1 for 30 min at 40°C, Amp2 for 15 min at 40°C, Amp3 for 30 min at 40°C, Amp4 for 15 min at 40°C, Amp5 for 30 min at room temperature and Amp6 for 15 min at room temperature. Between each step, ovaries were washed for 5×3 min with wash buffer at room temperature. For color detection, a working RED solution was made fresh by using a 1:60 ratio of Fast RED-B to Fast RED-A. One-hundred and fifty microliters of working solution was added to each tube containing ten pairs of ovaries, and color development was performed at room temperature for 8 min, following which ovaries were washed in PBT, counterstained with DAPI, and mounted in Vectashield as above. RNAscope experiments were repeated three times on control and *Wnt6-RNAi* ovaries, and once on ovaries of two lines expressing *dpp-RNAi* following the same protocol.

### BrdU feeding and staining

Two-day-old females were mixed with males and fed on filter paper soaked with 5% sucrose+10% yeast+10 mg/ml BrdU (Sigma B5002, diluted from a stock solution of 20 mg/ml in 20% ethanol) for three consecutive days at 18°C, changing to new vials of BrdU every day. Flies were then switched to cornmeal-molasses food (‘chase’) with fresh wet yeast paste as indicated. Ovaries were dissected in *Drosophila* Schneider's Medium, washed once with PBS, and fixed 15 min in 4% paraformaldehyde (Ted Pella) in PBS, followed by 15 min fixation in 4% paraformaldehyde in PBS+0.6% Triton X-100. Ovaries were washed twice with PBS+0.6% Triton X-100, and washed three times in DNase I buffer (66 mM Tris, pH 7.5, 5 mM MgCl_2_, 1 mM 2-mercaptoethanol, added fresh before use), 15 min each. Ovaries were treated with 50 units of DNase I (NEB, M0303S) in 0.5 ml DNase I buffer at 37°C for 30 min, washed with PBS containing 0.3% Triton X-100 and incubated overnight in rat anti-BrdU (BU1/75, OBT0030G, Bio-Rad) at 1:100. Ovaries were washed in PBS containing 0.3% Triton X-100, incubated in goat anti-rat IgG conjugated to Alexa Fluor 488 (Molecular Probes, used at 1:500) for 3 h at room temperature, washed and co-stained with DAPI.

### Fluorescence microscopy and imaging

All samples were imaged using a Zeiss Axio Imager M2 microscope equipped with an Apotome system and an AxioCam MRm camera (Zeiss). Samples were imaged using a 63×/1.4 oil Plan-Apochromat objective lens at room temperature. Projections of *z*-stacks were generated using the Orthoview function in the Zeiss Axiovision 4.8 software. Images were exported as 16-bit TIFF files and processed with Adobe Photoshop CS4.

### Counting and statistics

To determine the number of escort cells, ovaries containing the *PZ1444* reporter were stained with anti-β-galactosidase to label both the cap cell nuclei and the escort cell nuclei. Then, cap cells and escort cells were identified and counted separately based on their location and shapes of nuclei. For the quantification shown in Fig. 4E, anterior escort cells were identified by expression of *mCD8GFP* driven with *C587Gal4*, a plasma membrane-localized GFP, and by their location contacting the cap cells anteriorly. The numbers of GSCs were determined by anti-Hts staining, which labels the spectrosomes in GSCs and cystoblasts. Spectrosomes were identified and GSCs counted at the microscope rather than in 2D images, so that super-imposed spectrosomes were not mistaken for fusomes. GSCs were further identified by their attachment to cap cells, which were recognized by LamC staining, *PZ1444*, or their DAPI-stained nuclei. Student's *t*-test (two-tailed, two-sample equal variance) was used for statistical analysis and a *P*-value of <0.05 was considered significant.

## Supplementary Material

Supplementary information

Supplementary information
