# Supplementary material for: Wnt6 maintains anterior escort cells as an integral component of the germline stem cell niche
Source: Development. 2018 Feb 1;145(3):dev158527. doi: 10.1242/dev.158527 (PMC5818006; doi:10.1242/dev.158527)
Supplement: Supplementary information [file develop-145-158527-s1.pdf]

Fig. S1

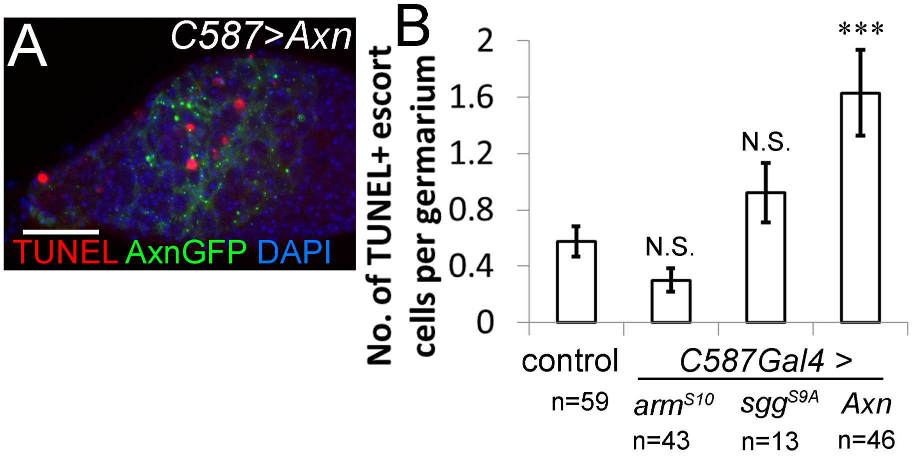

Figure S1. Quantification of the average number of TUNEL-positive escort cells after Wnt signaling is altered. (A) *Axin* overexpression in escort cells increases the number of TUNEL-positive nuclei in germaria (red). Overexpressed *Axin* is labeled with GFP (green). **(B)** Quantification of the number of TUNEL-labeled nuclei in each germarium. Escort-cell specific inactivation of Wnt signaling by overexpressing *Axin* increases the absolute number of TUNEL-labeled nuclei per germarium. Although activation of Wnt signaling with arm^{S10} or inactivation with sgg^{S9A} does not alter significantly the absolute number of TUNEL-labeled nuclei per germarium, this result is misleading because the total number of escort cells is altered. Fig. 1M in the main text shows that the percentage of TUNEL-labeled escort cells does significantly change in these cases. Flies contained both *C587Gal4* and *tubGal80ts* to drive escort cell expression at 29°C. Upon eclosion, flies were switched from 18°C to 29°C for 1 week before dissection. ***, p<0.001, Student's *t* test. N.S., not significant. Bar, 20 µm. n indicates the number of germaria counted for each experiment.

Development • Supplementary information

Fig. S2

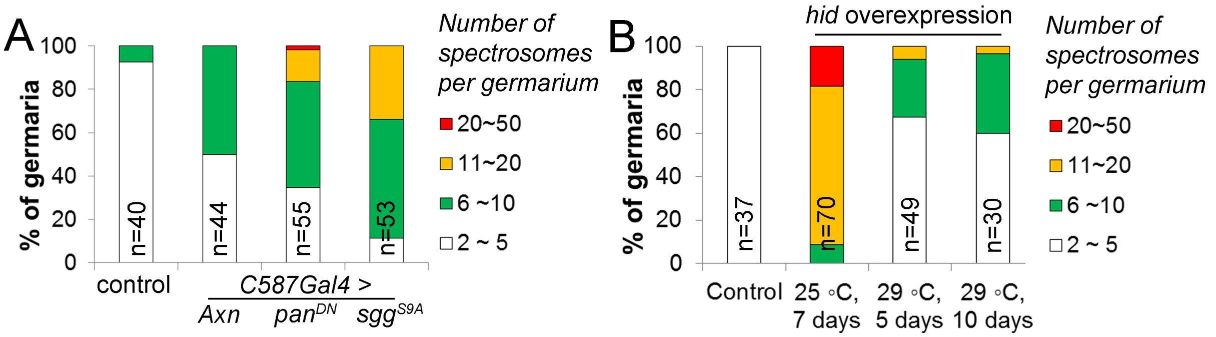

Figure S2. Tumor phenotypes result from reducing Wnt signaling in escort cells (A) or ablating escort cells by expressing the pro-apoptotic gene *hid* (B). Flies were under the control of *C587Gal4* and *tubGal80*^{ts} and were switched upon eclosion from 18°C to 29°C to induce Wnt signaling regulators **(A)**, or high expression of *hid* **(B)**; or switched from 18°C to 25°C to induce moderate expression of *hid* **(B)**. Germline differentiation defect was quantified by counting the number of germ cells that contain spectosomes (labeled with anti-Hts), which indicates an undifferentiated state. In (B), moderate expression of *hid* led to a greater differentiation defect than high levels of *hid*, probably because at higher *hid* levels, not enough escort cells remained to support the GSCs that give rise to undifferentiated spectrosome-containing cells. n indicates the number of germaria for each experiment.

Development • Supplementary information

Fig. S3

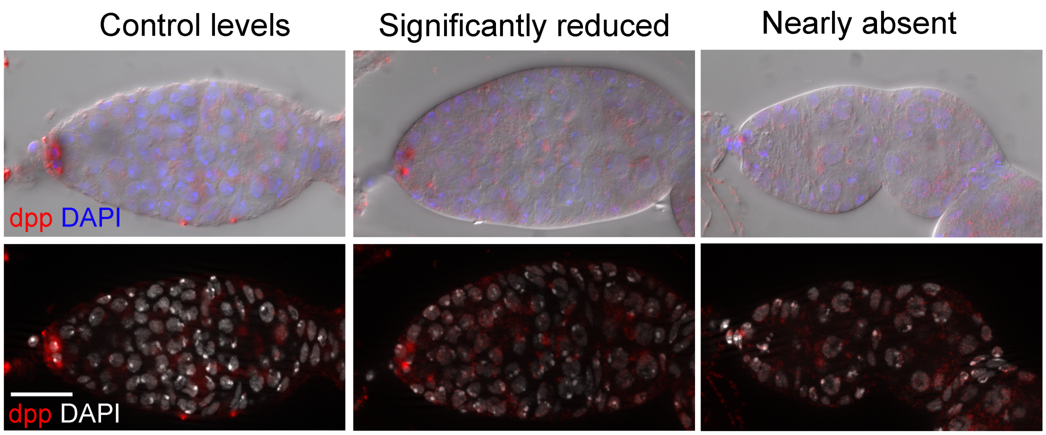

control	n=42	n=0	n=0
bab1>dpp RNAi (line #1)	n=6	n=12	n=1
bab1>dpp RNAi (line #2)	n=0	n=17	n=3

Figure S3. ***In situ*** **hybridization for** ***dpp*** **with RNAScope is specific for** ***dpp*****.** As expected, the *dpp* signal is observed in cap cells in controls (42/42). For negative controls, *dpp* was knocked down in cap cells with either of two different RNAi lines. Compared to controls (left images), knockdown samples were observed to have a reduced (middle images) or nearly absent (right images) signal. The frequency of these classes is shown below each image. These data show that RNAi line #2 is a stronger line with more penetrant knockdown than RNAi line #1, and that the RNAscope *dpp* signal is specific to *dpp*. Flies were under the control of *tubGal80^{ts}* and were switched upon eclosion from 18°C to 29°C for 2 days.

Fig. S4

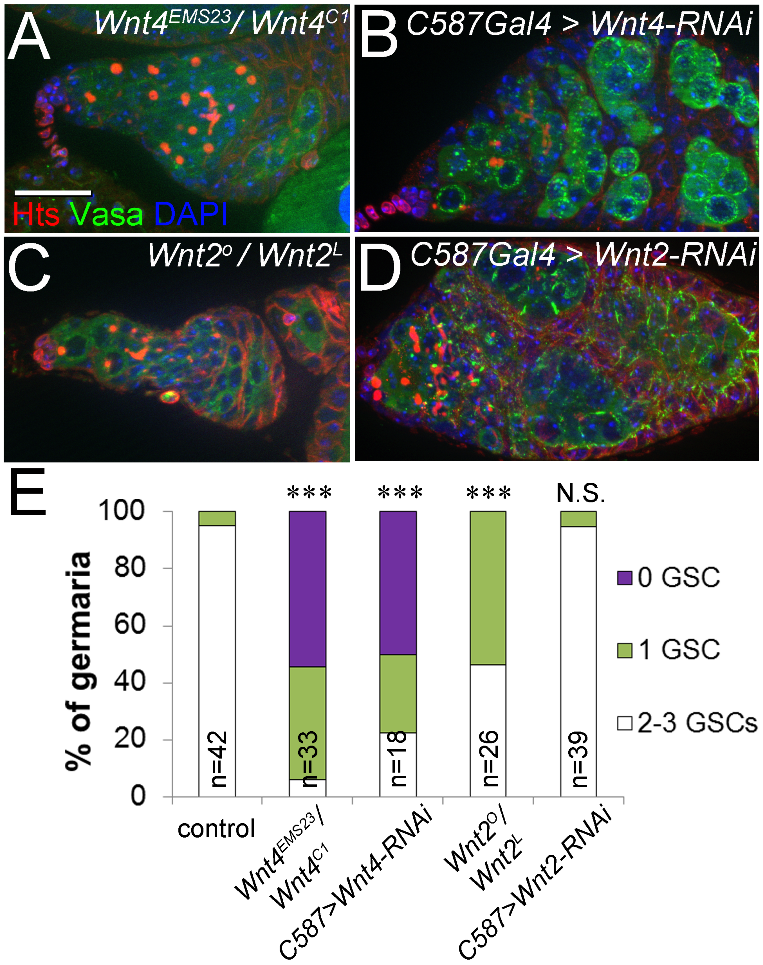

Figure S4. Autocrine Wnt4 ligand in escort cells is required for GSC maintenance. (A-C) GSC loss was observed in *Wnt4* loss-of-function mutant, *Wnt4* RNAi driven by escort cell-specific *Gal4* (*C587Gal4*) and *Wnt2* loss-of-function mutant. **(D)** *Wnt2* RNAi in escort cells caused germline encapsulation defect, but did not induce GSC loss. Hts (red) labels spectrosomes and fusomes, and Vasa (green) labels the germline. **(E)** Quantification of GSC numbers in control germaria and the genotypes in (A-D). Statistical analysis was performed on the average number of GSCs. N.S., not significant; ***, $p<0.001$, Student's *t* test. Bar, 20 µm. Flies were under the control of *tubGal80*^{ts} and were switched upon eclosion from 18°C to 29°C for 10 days. n indicates the number of germaria counted for each experiment.

Development • Supplementary information

Fig. S5

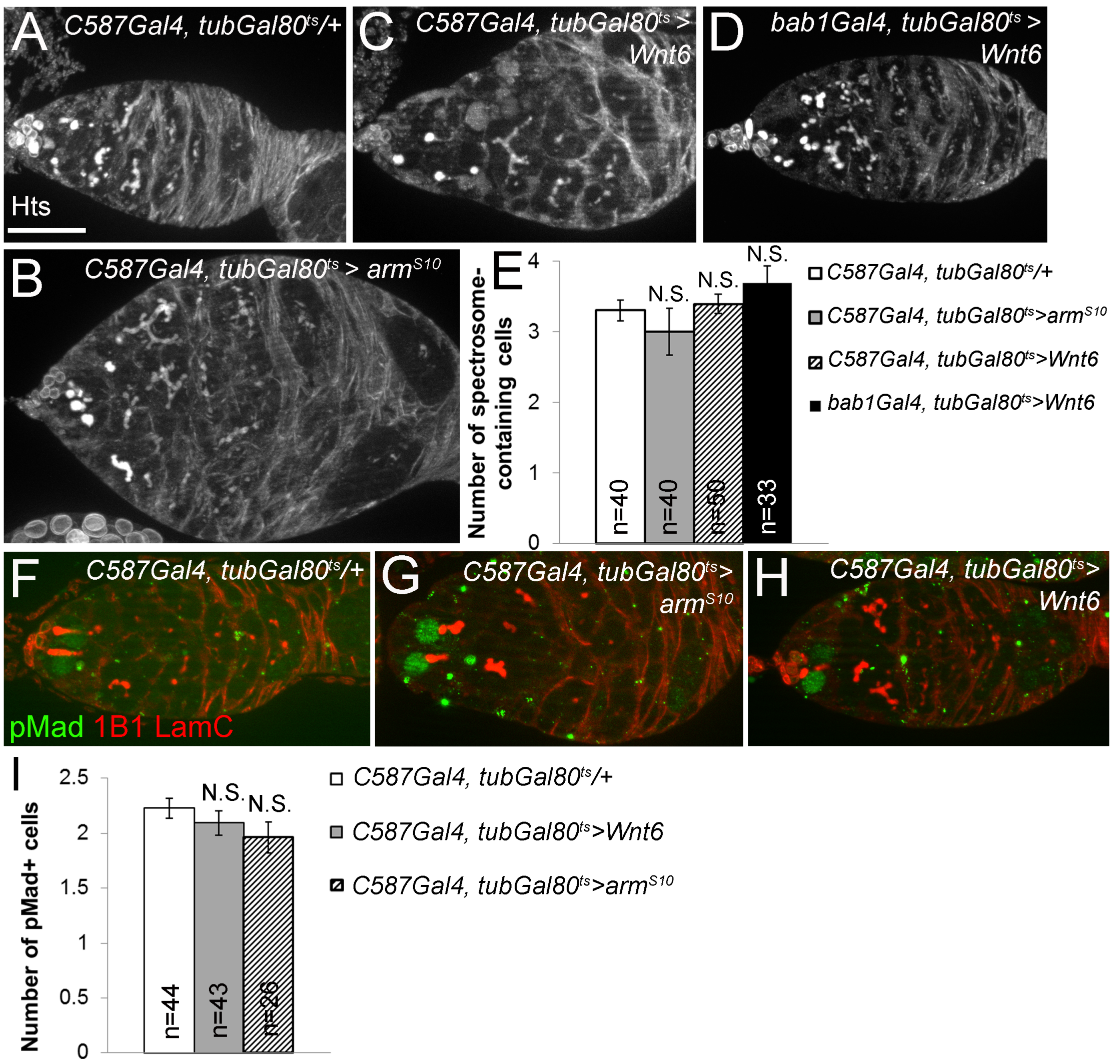

Figure S5. Wnt6 or Wnt signaling is not sufficient to maintain excess GSCs. (A) Control germarium, labeled with Hts antibody to show spectrosomes and fusomes. **(B)** Adult-onset constitutively active Wnt signaling, caused by expressing *arm*^{S10} in escort cells with *C587Gal4*, is not sufficient to maintain excess GSCs in the niche. The germarium is enlarged with excess escort cells. Follicle-cell encapsulation defects are also observed, but germline differentation is normal. **(C-D)** *Wnt6* overexpression from escort cells with *C587Gal4* (C) or cap cells with *Bab1Gal4* (D) is not sufficient to maintain excess GSCs in the niche. **(E)** Quantification of spectrosome-containing GSCs in conditions (A-D). **(F-H)** Similar to A-C, except that GSCs are labeled by pMad staining as well as Hts/1B1 staining. Overexpression of activated *arm*^{S10} or *Wnt6* in escort cells is not sufficient to maintain excess GSCs in the GSC niche. **(I)** Quantificaiton of F-H. N.S., not significant, Student's *t* test. Flies were under the control of *tubGal80*^{ts} and were switched upon eclosion from 18°C to 29°C for 10 days. n indicates the number of germaria counted for each experiment.

Development • Supplementary information

Fig. S6.

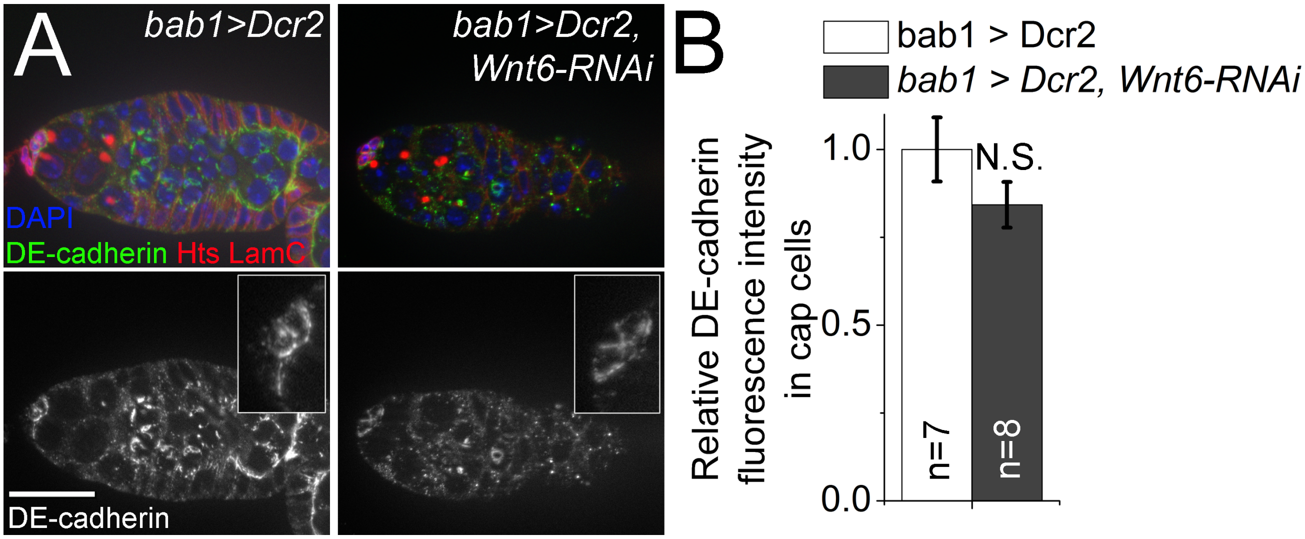

Figure S6. *Wnt6-RNAi* in cap cells does not affect DE-cadherin levels between cap cells and GSCs. (A) DE-cadherin levels are similar at the junctions between cap cells and GSCs in controls and in germaria with *Wnt6* knocked down in cap cells. Cap cells are identified by anti-LamC, which labels the nuclear envelope of the cap cells, and GSCs are identified by their spectrosomes labeled with Anti-Hts and their attachment to the cap cells. *bab1Gal4* is a cap cell –specific driver. **(B)** Quantification of the relative fluorescence intensity of DE-cadherin in the cap cells of control and *Wnt6* RNAi germaria. N.S., not significant, Student's *t* test. Bar, 20 µm. Flies were under the control of *tubGal80*^{ts} and were switched upon eclosion from 18°C to 29°C for 10 days. n indicates the number of germaria counted for each experiment.

Development • Supplementary information

Fig. S7.

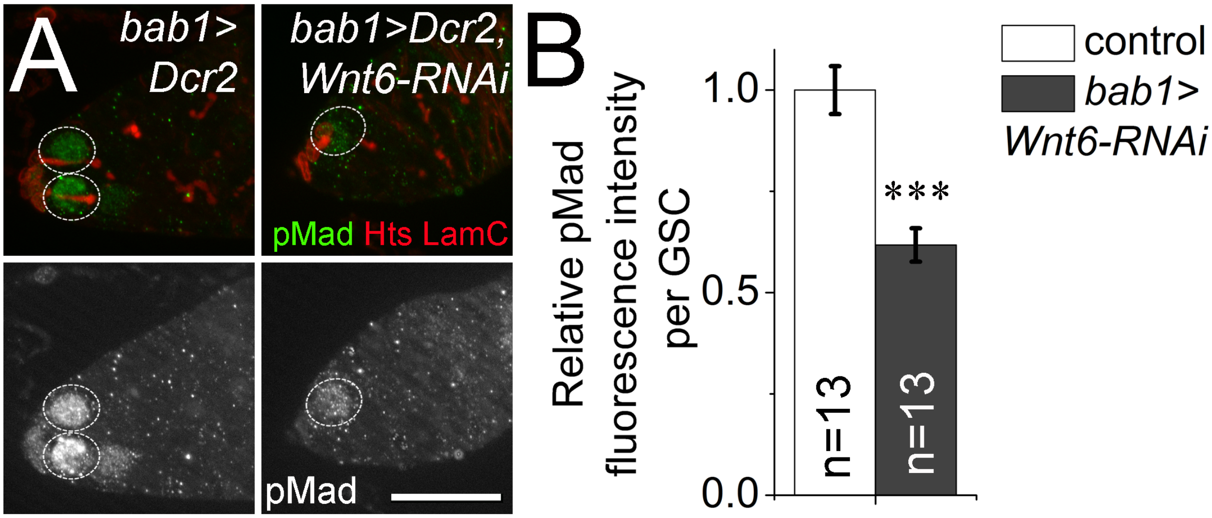

Figure S7. *Wnt6* RNAi in cap cells reduces BMP signaling levels in GSCs. (A) pMad staining was used as a marker for BMP signaling activity. Cap cells are identified by anti-LamC, and GSCs are identified by their spectrosomes labeled with anti-Hts and their attachment to the cap cells. **(B)** Quantification of the intensity of pMad staining in GSCs residing in the GSC niche (outlined by dashed circles) in (A). ***, p<0.001, Student's t test. Bar, 20 µm. Flies were under the control of *tubGal80^{ts}* and were switched upon eclosion from 18°C to 29°C for 10 days. n indicates the number of germaria counted for each experiment.

Fig. S8

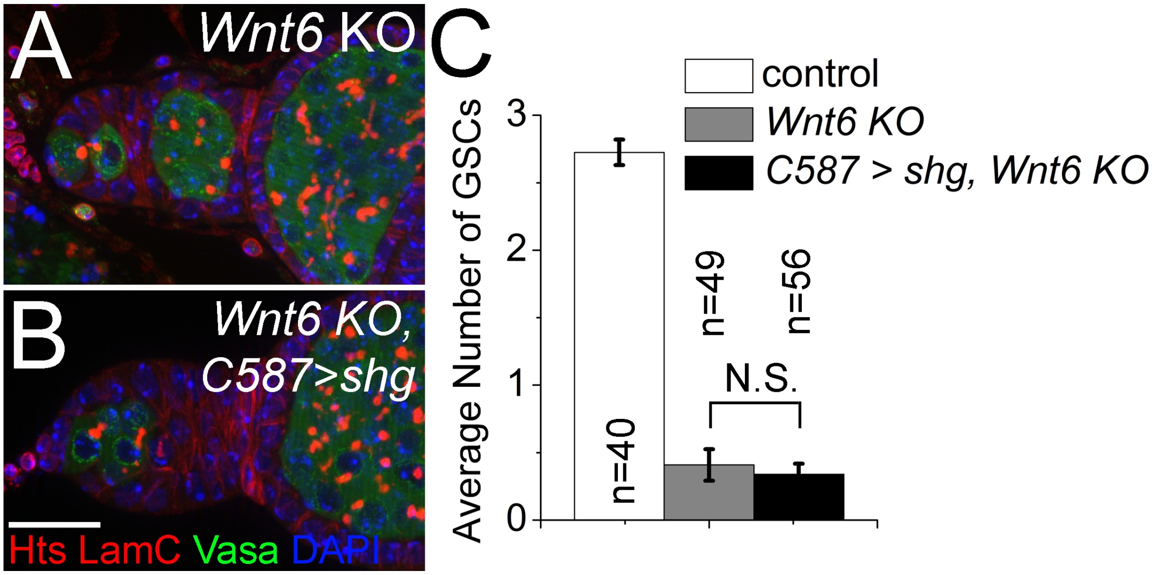

Figure S8. Forced expression of DE-cadherin (*shg*) in escort cells does not suppress GSC loss in *Wnt6* knock-out germaria. (A,B) *Wnt6* knock-out germaria exhibited GSC loss, which was not alleviated by *shg* expression driven in the escort cells with *C587Gal4*. **(C)** Quantification of GSC numbers in control germaria and genotypes in A-B. Statistical analysis was performed on the average number of GSCs. N.S., not significant, Student's *t* test. Bar, 20 µm. Flies were under the control of *tubGal80*^{ts} and were switched upon eclosion from 18°C to 29°C for 10 days. n indicates the number of germaria counted for each experiment.

Development • Supplementary information

Fig. S9

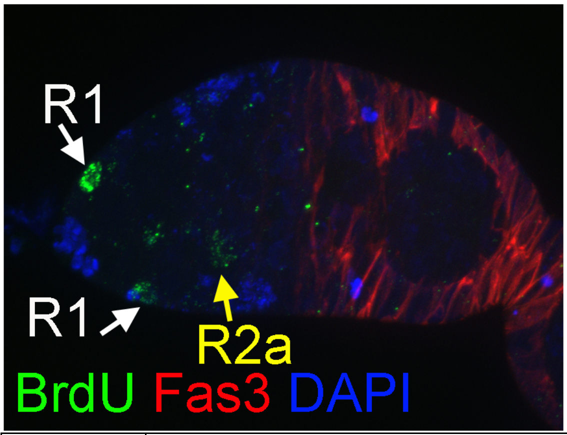

	No. of BrdU-labeled ECs in each region			
	**Region 1**	**Region 2a**	**Region 2a/2b**	**Total germaria**
Exp. 1	3 ECs	12 ECs	3 ECs	88
Exp. 2	2 ECs	2 ECs	1 EC	55

Figure S9. BrdU-labeled escort cells appear to migrate anteriorly. Control female flies were fed BrdU for 3 days at 18°, then allowed to feed on normal food ("chased") for 16 days. Ovaries were dissected and stained for BrdU. Because cell cycling is only observed by FUCCI in Regions 2a and 2a/2b, the appearance of BrdU-labeled escort cells in Region 1 suggests that these cells moved anteriorly after being labeled. Germaria from flies not chased with normal food or chased for shorter periods contained BrdU labeled germline cells that obscured the appearance of BrdU-labeled escort cells (not shown).

Development • Supplementary information
